# Does an Incidental Meckel's Diverticulum Warrant Resection?

**DOI:** 10.7759/cureus.10307

**Published:** 2020-09-08

**Authors:** Shermeen Rahmat, Prerna Sangle, Osama Sandhu, Zarmeena Aftab, Safeera Khan

**Affiliations:** 1 Internal Medicine, California Institute of Behavioral Neurosciences and Psychology, Fairfield, USA; 2 Family Medicine, California Institute of Behavioral Neurosciences and Psychology, Fairfield, USA

**Keywords:** incidental meckel's diverticulum, resection

## Abstract

Meckel's diverticulum (MD) is the most common gastrointestinal malformation. The management of symptomatic Meckel's diverticulum has been undecidedly resection; however, the management of incidental Meckel's diverticulum has been fraught in comparison. As a systematic literature review, PubMed, PubMed Central (PMC), and MEDLINE were used. The search phrase utilized was "Meckel Diverticulum/Surgery [Mesh]" and resection incidental. The search was completed on July 18, 2020 and was limited to 1980 until the day of the search. Searches resulted in 62 initial articles on PubMed. On initial screening, 23 of these articles met the criteria. The references of these 23 articles were screened for relevant studies, yielding a total of 31 studies of which all were assessed for quality. Four articles made a recommendation for no resection. Twelve studies made a recommendation for resection. Ten studies concluded that resection should be completed in the presence of risk factors. Lastly, five studies made no clear recommendation. In recent literature, there has been a shift towards resection for all or in those with high-risk factors. In the future, it will be necessary for researchers to determine if resection is recommended for all patients with incidental MD or in those with risk factors. If only in those with risk factors, it will be important that research is completed to create evidence-based guidelines to support the risk factors.

## Introduction and background

**Figure 1:**
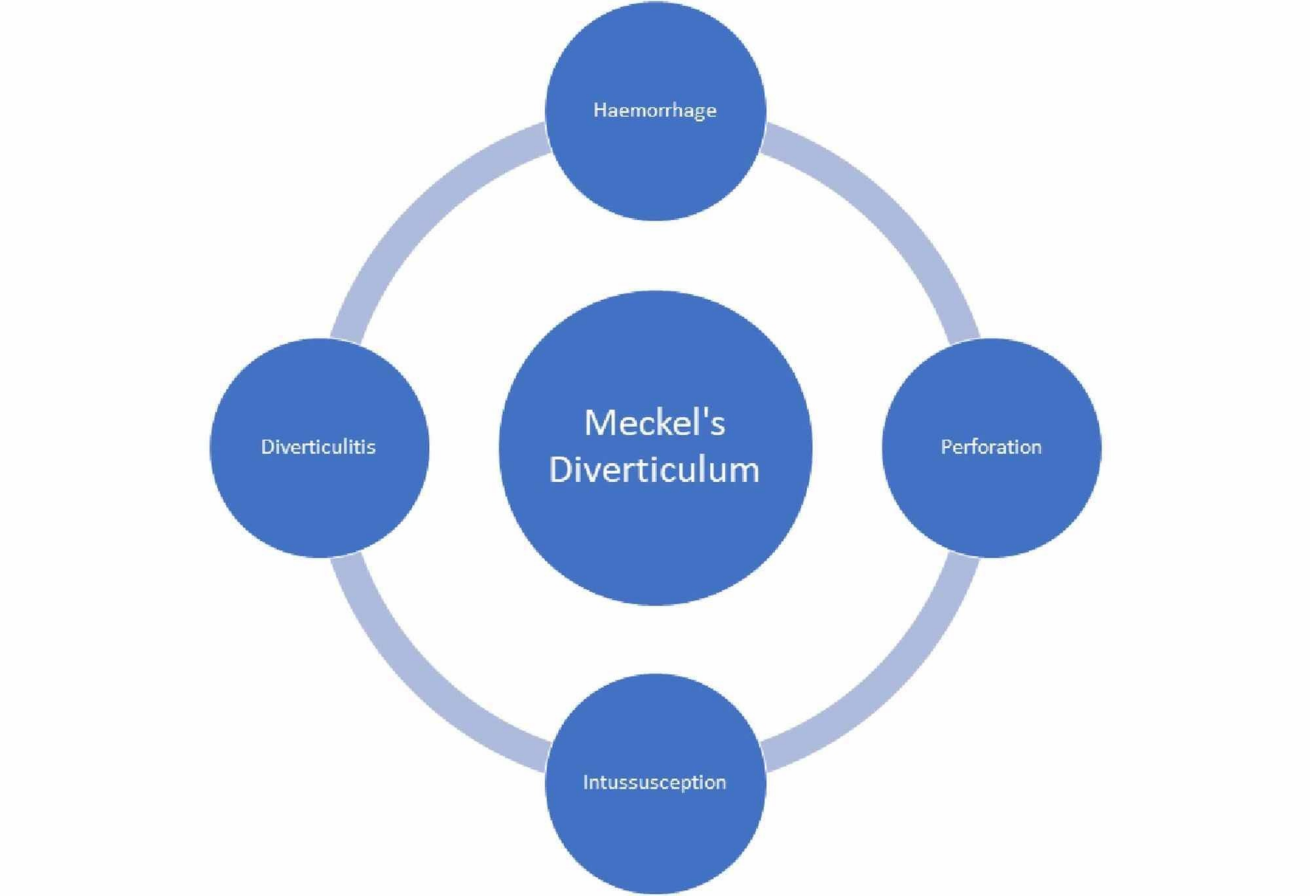
Common Complications of Meckel's diverticulum (MD)

Meckel's diverticulum (MD) is a congenital abnormality due to the failure of the vitelline duct to close. It is the most common congenital gastrointestinal malformation, with studies demonstrating it is present in approximately two percent of the population [1]. It is a true diverticulum containing all three layers of the small bowel. The complications of Meckel's diverticulum have been well documented. These include most commonly obstruction, followed by hemorrhage, perforation, diverticulitis, and intussusception [2]. These are highlighted in Figure 1. 

A worrisome feature of Meckel's diverticulum is the propensity to develop cancer, particularly malignant carcinoid tumors [3]. MD may also contain heterotopic gastric mucosa, which may lead to substantial rectal bleeding [4]. In patients presenting with symptomatic MD, there is a higher incidence in males compared to females, with a 1.5-4:1 ratio. Symptomatic MD is more common in those that are younger. Symptomatic MD are treated with resection [5]. Resection of an incidental MD is more controversial than symptomatic MD [6].

Most of the research done in terms of Meckel's diverticulum has been retrospective. There has been a paucity of randomized control studies completed concerning all complications associated with MD and none in association with incidental MD. The majority of studies focused on single centers. There is difficulty in developing a randomized control trial because the length of follow up required is extensive, and the invasive nature of the procedure. Research in the area has been rather continuous, with one or two articles published regularly about all aspects of Meckel's diverticulum [7].

Though research on the general area of MD has been regular, there is still a lack of information on the management of incidental MD. Therefore, Meckel's diverticulum still poses a dilemma due to the lack of information in regard to the likelihood of complications of a silent MD versus the possibility of complications due to surgery. These two outcomes should be weighted appropriately to develop a suitable evidence-based guideline. Multiple factors may impact the decision for resection. These factors are highlighted by Robijn et al.'s 2006 risk assessment tool, which works to take into account all the factors that increase the risk of complications of a silent MD such as male gender, individuals aged less than 45, presence of a fibrous band and the length of the diverticulum especially if longer than two centimeters [8]. 

The importance of gathering and analyzing this information is to add clarity and improve management, which in turn will either prevent unnecessary surgical intervention or prevent the myriad of complications associated with MD. It is worthwhile to determine the necessity of intervention to prevent iatrogenic harm. Therefore, this systematic review aims to examine the previous literature and elucidate if an incidental Meckel's diverticulum warrants resection in both children and adults.

## Review

Method

The search sources utilized were PubMed, PubMed Central (PMC), and MEDLINE. The search phrase used was "Meckel Diverticulum/Surgery [Mesh]" and resection incidental. The search was completed on July 18, 2020 and was limited to 1980 up until the day of the search. Duplicate articles were removed using the respective search resources. The identified articles were then screened for their relevance, and selected articles were read in full. Articles in which the abstract contained introduction, methods, results, and conclusion were included even when the full article was not available. Of the articles selected, the associated references were screened for relevant articles. Articles that were selected were then quality screened using A MeaSurement Tool to Assess systematic Reviews (AMSTAR) and New Castle Ottawa Tool [9,10]. The inclusion criteria for this systematic review included: relevancy to the topic, English or German language, and post-1980. The exclusion criteria were case series with less than four patients and editorials. A results table was compiled; the result of each study was included with its recommendation about resection of a Meckel's diverticulum. The Preferred Reporting Items for Systematic Reviews and Meta-Analyses criteria (PRISMA) statement for systematic review was applied to the development of the design, search, and writing of this paper [11].

Results

**Figure 2:**
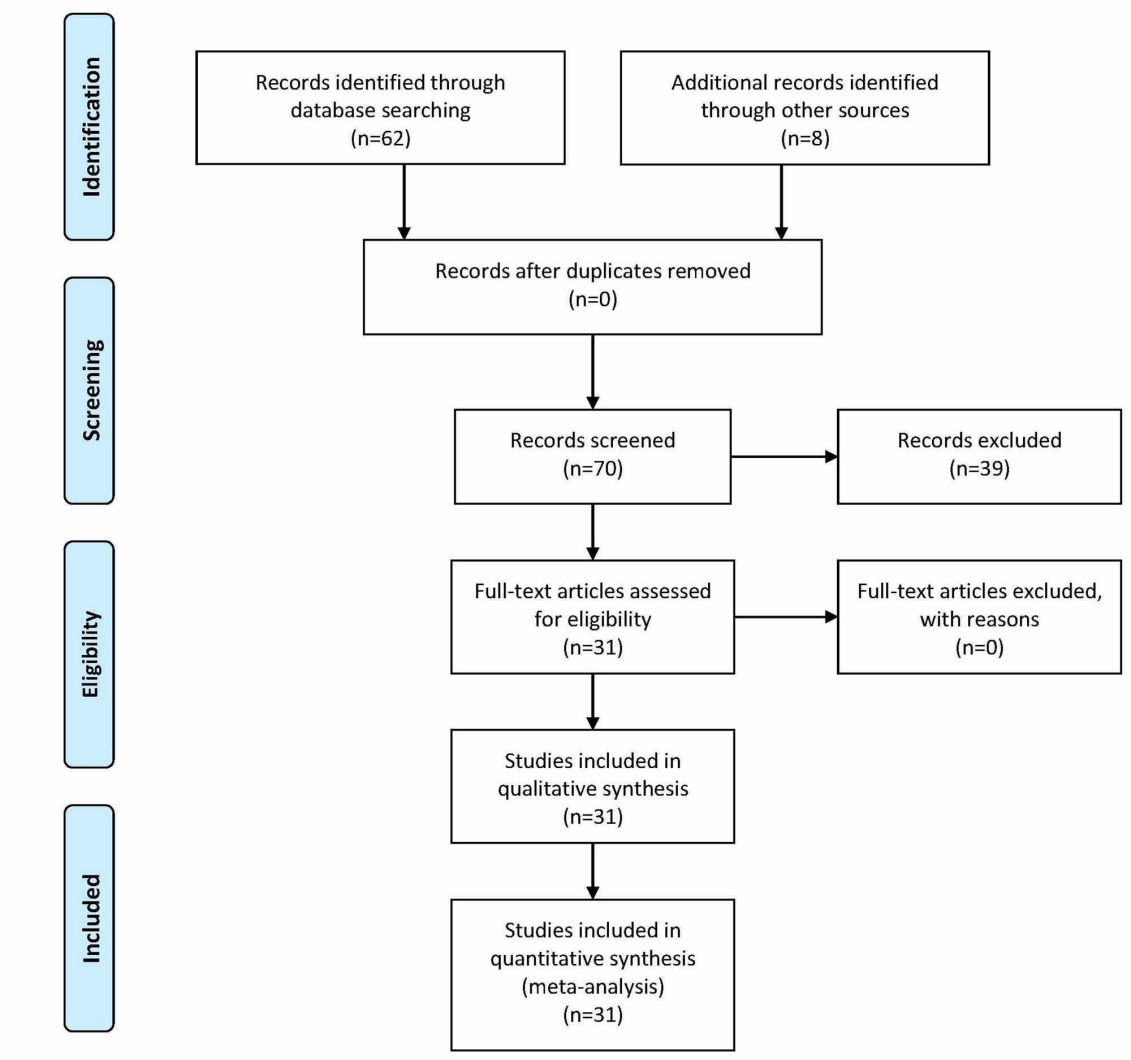
Preferred Reporting Items for Systematic Reviews and Meta-Analyses criteria (PRISMA) Flow Chart Figure adapted from [11].

The key terms resulted in 62 initial articles on PubMed. On initial screening, 23 of these articles met the criteria. The references of these 23 articles were also screened for relevant studies yielding a total of 31 studies of which all were assessed for quality using the above systems. Figure 2 depicts this flow [11]. 

Eleven of the papers were either systematic reviews or traditional reviews. Twenty of the papers were observational studies. None of the studies were prospective; therefore, all were retrospective, most commonly retrospective cohort studies. 

Unfortunately, many of the studies, particularly the systematic reviews, did not mention the total number of patients included or individual articles assessed; however, of the studies that did mention the number of patients in total that were tallied was 5,310.

The results of each study were broken down into four categories: no clear recommendation, no resection, resection, and resection with specific risk factors or conditions. Table 1 illustrates the four categories and the number of studies within each. 

**Table 1:** Outcome of studies.

Outcome	Number of Studies
No Clear Recommendation	5
No Resection	4
Resection	12
Resection with risk factors	10

Five articles made no explicit recommendation of which two delineated factors that should not be used to make the decision for or against resection. Ten studies made a case for resection when certain circumstances were present. These circumstances were factors that were likely to increase complications in the future due to Meckel's diverticulum. Twelve articles made a case for resection. The remaining four articles recommended no resection for incidentally found Meckel's diverticulum.

Discussion

We conducted this systematic literature review of all studies relating to the management of incidentally found Meckel's diverticulum after the year 1980. The articles were broken down into their conclusion with relation to resection. The four categories and their relevant articles are compared and contrasted in the following sections, with a final discussion about limitations.

No Clear Recommendation

Of the total 31 articles, five made no clear recommendations on the management of incidental MD. One of these articles, Varcoe et al., does not make a clear distinction on the management of MD, but does highlight factors that should be considered. The article underlines how heterotopic gastric mucosa is not a reliable indicator in the decision-making process for resection [4]. Lohsiriwat et al., which also made no explicit recommendation to the resection of silent MD but demonstrated MDs that become symptomatic are more likely to be longer, alongside a higher rate of ectopic mucosa [12]. These two articles highlight the general level of confusion about many of the risk factors associated with MD. 

Hansen et al.'s study, a comprehensive systematic literature review covering epidemiology, presentation, and management of MD, concludes that resection of an incidental MD remains contentious. The authors do consider various studies to highlight the fraught nature of the literature, but at the same time, the authors place forward their opinion. In the pediatric population MDs that are recognized at the time of surgery, Hansen et al. recommend resection. In the adult population, resection is recommended if the MD has a length greater than two centimeters. In the last cohort, elderly individuals, the authors recommended against resection. Hansen et al. underline the importance of the initial procedure and the graveness of the situation to determine if resection should be attempted. This balanced approach is further echoed in section four, but Hansen et al. compare the literature and highlight how the literature itself is conflicting [5].

Sagar et al., a systematic review, makes no clear recommendation much like Hansen et al. demonstrates the overall nature of literature is conflicting, but also defaults to Park et al. [13,5,14]. Park et al. is one of the most extensive retrospective studies completed with 1476 patients and recommends resection in certain circumstances for incidental MD [14]. 

In the management of incidental MD, a few studies, highlighted by Table 2, demonstrated no clear recommendation. Even within the few studies that concluded no specific approach, both Sagar et al. and Hansen et al. demonstrated that regardless of the overall controversial nature of the literature, certain studies withstand scrutiny to a higher degree and should be used as guidance [13,5]. 

**Table 2:** Studies that made no clear recommendations for or against resection. MD: Meckel's diverticulum, HGM: heterotopic gastric mucosa

Author	Year of Publication	Purpose of the Study	Type of Study	Result/Conclusion
Hansen et al. [5]	2018	To review recent literature concerning MD	Systematic Review	No Clear Recommendation.
Lohsiriwat et al. [12]	2014	Compare characteristics of MD removal from asymptomatic patients & symptomatic patients.	Observational Study [84 Patients]	No clear recommendation, but highlighted that symptomatic patients had longer MD and more likely to have ectopic tissue.
Sagar et al. [13]	2007	Complications regarding MD in adults	Systematic Review	No clear recommendation but refers to the Mayo Study
Varcoe et al. [4]	2004	Should the choice of resection technique depend on the macroscopic appearance of the MD	Observational Study [77 Patients]	HGM is an unreliable indicator to aid resection decisions. Simple transverse resection is not recommended for the short Meckel's diverticulum
Lang- Stevenson et al. [15]	1983	Determine if incidental MD should be resected	Literature Review and Expert Opinion	Recommends further research be undertaken

Recommends No Resection for Incidental MD 

Only four studies, as highlighted in Table 3, recommended no resection. Bona et al. and Zani et al., both published in 2008, and both literature reviews support no resection [6,16]. Bona et al. covers the incidental finding of a carcinoid tumor in an asymptomatic MD during an inguinal hernia repair and conducts a literature review of the management of asymptomatic MD. This underlines the possibility of silent malignancy that would be prevented if asymptomatic MD were resected, but the study highlights how overall the literature recommends against resection [6]. The study is uniquely placed as it highlights how previous research and literature only examined laparotomy. However, not many studies have been done comparing outcomes of a laparoscopic approach, and the associated outcomes of a MD left in-situ. Bona et al., in particular, highlight this is the case with Zani et al. [6,16]. 

**Table 3:** Recommend no resection of incidental MD. MD: Meckel's diverticulum

Author	Year of Publication	Purpose of the Study	Type of Study	Result/Conclusion
Bona et al. [6]	2008	Management of unexpected MD in abdominal operation	Literature Review and Case Report	No compelling evidence in the literature to recommend prophylactic diverticulectomy
Zani et al. [16]	2008	To establish the prevalence, morbidity, and mortality due to MD.	Systematic Review	The evidence does not support the resection of incidentally detected MD.
Stone et al. [17]	2004	Present experience over the past ten years with MD	Observational Study [47 patients]	Incidental removal of asymptomatic diverticula, particularly in women, is not recommended
Peoples et al. [18]	1995	Should incidental MD be resected?	Observational Study [90 patients]	Incidental diverticulectomy in adults should be abandoned.

Zani et al. demonstrate that postoperative complications are increased (5.3%) compared to MD that is left in-situ (1.3%). According to this study, the number needed to treat to prevent one death would be 758. The article makes the argument the pediatric population should be included in those that are no longer operated upon for asymptomatic MD, even though the mortality is highest in the pediatric group emphasizing the still low rate of mortality even in this cohort [16]. 

Stone et al., an observational study of a single center with 47 patients, demonstrates at their center resection was not supported, especially in women, who were less likely to become symptomatic. The study was predominantly women; however, men in most studies tend to predominate. There was low mortality with both symptomatic resection and asymptomatic resection, but both had complications of morbidity of approximately eight percent. Both the small number of patients in this study and the disproportionate number of women may cause difficulty in applying these study results to a broader number of patients. Alongside, three of the four patients that had complications in the study underwent ileal resection; therefore, the relation between diverticulectomy and morbidity is not clear [17]. 

Peoples at al. mirrors much of Stone et al. Peoples at al. was an observational study of 90 patients analyzing data from five years from 1989 to 1993. The study compares their morbidity and mortality for incidental MD compared to other studies. The results calculated for procedures only completed for symptomatic MD the morbidity and mortality would be 0.2% and 0.04%, but the risks for resecting all asymptomatically discovered were 4.6% and 0.2% [18]. Though both Peoples et al. and Stone et al. highlight the increased rate of morbidity, the two studies have hugely varying rates of morbidity 4.6% and 8% [17,18]. This highlights a discrepancy between morbidity. The difference could be accounted for through the small sample sizes in both studies.

Recommends Resection for Incidental MD 

Under this heading falls the largest number of studies of which there are 12. Seven of the 12 articles have been published within the last 10 years. The increasing number of studies underline that asymptomatic MD should be removed; therefore, a paradigm shift may be beginning to occur in terms of the controversy related to the management of asymptomatic MD.

Matsagas et al., published in 1995, had the largest number of patients involved in the topic - 2074 patients [19]. The article recommended resection, regardless of age. Multiple studies contrasted the mortality and morbidity associated with Peoples et al. and Stone et al. [18,17]. Cullen et al. determine that the rate of postoperative complications from resection of an incidental MD is lower than symptomatic resection and that asymptomatic resection has associated lower morbidity and mortality than surgical resection in those with symptomatic MD. It concluded that the lifetime risk of complications from MD was 6.4%, while those of surgery was 1% and 2% concerning mortality and morbidity rates. Therefore, benefits outweigh the risk in terms of morbidity and mortality and asymptomatic diverticulum should be resected [20]. Loh et al. also support that resection of incidental MD had fewer complications compared to symptomatic resection [21].

Another complication associated with MD is that it increases with age in contrast to the others - malignancy. Thirunavukarasu et al. highlight this risk. The incidence of Meckel's diverticulum related malignancy increased with age, alongside that there was a 70-time increased risk of neoplasm in MD compared to other ileal sites [3]. Mora-Guzman et al. build upon this concept highlighting how, because asymptomatic MDs are not always resected. However, then neuroendocrine tumors may be more common than historically thought [22].

One area of contention is if resection should occur in all individuals or on a case-by-case in those with risk factors. Thirunavukarasu et al., once again, highlights a critical reason that not only the pediatric should have incidental MD resected [3]. Gezar et al. underline a unique concept that in their observational study, the macroscopic appearance of MD could not be used to determine the likelihood of heterotopic gastric mucosa; therefore, it should not be used to determine if surgery is required [23]. At the same time, the study highlighted how arbitrary cut-of-values of measurement such as length, diameter, and base and their association of increased risk of complications was somewhat fraught with difficulty because it was observed that these factors increase with increasing age [23]. The 12 studies are listed in Table 4.

**Table 4:** Studies that recommend resection of incidental MD MD: Meckel's diverticulum

Author	Year of Publication	Purpose of the Study	Type of Study	Result/Conclusion
Mora-Guzman et al. [22]	2019	Determine if incidental diverticula should be removed.	Observational Study [66 Patients]	Resection of incidentally found MD
Demirel et al. [24]	2019	Evaluate risk factors that cause complications of MD	Observational Study [62 Patients]	The existence of ectopic mucosa does not affect the development of complications rate requiring urgent surgery. Recommend Resection
Chen et al. [25]	2018	Review experiences and management strategies	Observational Study [286 Patients]	Safe and feasible to remove incidentally found MD.
Gezer et al. [23]	2016	Does macroscopic appearance correlate with clinical features, histopathological findings, future complications, and management decisions?	Observational Study [50 Patients]	Incidentally detected MD should be removed regardless of its macroscopic appearance.
Loh et al. [21]	2014	Assess the Safety of the resection of symptomatic and asymptomatic MD concerning postoperative complications	Literature Review	Resection of incidental MD can be recommended in patients in cases without contraindications (peritonitis, cancer, ascites, or immunosuppression).
Thirunavukarasu et al. [3]	2011	Assess epidemiology and risk of MD cancer (MDC) and compare it with other ileal malignancies.	Observational Study [163 Patients]	Incidental MD is best treated with resection
Caracappa et al. [26]	2014	Should prophylactic resection be recommended?	Literature Review and Case Report	Recommend excision.
Zulfikaroglu et al. [27]	2008	Compare characteristics, morbidity, and mortality of incidental and symptomatic MD.	Observational Study [76 patients]	Resection of an incidentally found MD is not associated with increased operative morbidity or mortality.
Chiu et al. [28]	2000	Comparison of clinical picture associated with diverticula at different parts of the small bowel.	Observational Study [88 Patients]	The small bowel diverticula, except for Meckel's diverticulum, did not need to be treated if there were no significant symptoms.
Matsagas et al. [19]	1995	Determine the incidence of MD, the correlation between the histopathology and presentation. Review experiences	Observational Study [2074 Patients]	Resection regardless of age.
Cullen et al. [20]	1994	Determine if MD discovered incidentally at operation should be removed.	Observational Study [58 Patients]	MD incidentally recognized during operation should be removed for most patients, regardless of age.
Kovarik et al. [29]	1981	Should abdominal exploration routinely include the search for MD in asymptomatic patients?	Observational Study [13 Patients]	MDs constitute a significant threat to the future well-being of a patient. Incidental removal is associated with minimal risk of complications.

Recommends Resection of Incidental MD in High-Risk Patients 

The number of studies that recommend resection of all incidental MD or those that recommend resection in high-risk individuals recently published highlights a change in trend compared to earlier decades. The present discussion has shifted to if all incidental MD should be resected or in those with high risk. Many of the studies have similar findings in terms of individuals and traits that increase risk. It is possible this shift is due to increased concern in relation to malignancy, alongside the use of laparoscopic techniques which may demonstrate less morbidity, and better characterization of the long-term complications of MD. 

Park et al. is the second-largest study completed with 1476 patients over the years 1950 to 2002. The study determined that if patients with incidental diverticulum fulfilled the criteria, they should be prophylactically removed. Four criteria determined were: patient age <50 years, male sex, diverticulum less than two cm, and the presence of histologically abnormal tissue. The higher number of criteria met, the more likely the patient will develop asymptomatic MD [14]. Other studies mirror these criteria, but with slight differences such as in age, Lequet et al. and Mckay et al. have an age cut off of 50, while Robijin recommends in those less than 45 years old [30,31,8]. A few studies have the presence of a fibrous band [8,32]. 

Park et al. define the pediatric population as those less than 11 years old; this separation was useful in highlighting the different presentation of those under 11 and those over 11, obstruction, or bleeding, respectively [14]. Also, within the pediatric population, Onen et al. and St-Vil et al. highlight children under eight or "early childhood" should have resection of asymptomatic MD [33,34]. These studies contrast Park et al. and where the median age of symptomatic diverticula was 27, and the mean age was 31 [14]. Lindeman et al., a newly published literature review, underlines much of the issues highlighted before: that several studies focused on laparotomy, the increased risk of cancer, and the increasing number of studies supporting a risk-based assessment [34], and also selects to support a risk-based approach. It illustrates the importance of now defining the mortality and morbidity associated with laparoscopy.

Overall, within this sub-category, there is a general alignment that those with increased risk should have a resection. Overall, the criteria between many of the studies are similar with slight deviations in values. These can be seen in Table 5. 

**Table 5:** Studies that recommend resection with risk factors present. MD: Meckel's diverticulum

Author	Year of Publication	Purpose of the Study	Type of Study	Result/Conclusion
Lindeman et al. [34]	2020	Review how MD may present, either symptomatically or as an incidental finding.	Literature Review	No consensus on the treatment. Patient-bound characteristics used in decision-making.
Lequet et al. [30]	2017	The aim is to describe the circumstances in which MD is diagnosed, indications, and modalities of surgical treatment.	Literature Review	Surgery is not indicated for the complications if there are no risk factors: male gender, age < 40, MD < 2 cm, and the presence of macroscopically mucosal alteration.
Mckay et al. [31]	2007	Aim to outline indications for resection of incidental MD.	Observational Study [29 Patients]	Resection in those less than 50
Robijin et al. [8]	2006	Assess the complications of a non-resected MD and compare against resection to weigh in and justify prophylactic resection	Literature Review	The Risk Score is based on four risk factors: male sex, patients < 45 years, MD >2 cm, and the presence of a fibrous band. Resection of asymptomatic MD with a Risk Score of > or = 6 points.
Park et al. [14]	2005	Determine which diverticula should be resected following incidental finding during abdominal surgery.	Observational Study [1476 Patients]	Recommend removal when the following are present were patient age <50 years. Male sex, diverticulum >2 cm, and the presence of histologically abnormal tissue.
Onen et al. [33]	2003	Determine the morbidity and mortality of MD. Evaluate patient age, MD complications, and postoperative complications.	Observational Study [74 Patients]	Resection recommended in all children younger than eight years in the absence of absolute contraindication.
Groebli et al. [35]	2001	Evaluation of the effectiveness of various investigations and criteria for removing asymptomatic diverticula.	Observational Study [119 Patients]	Criteria for resection: male sex, age <40, ASA score, the procedure being done, the size and position of the diverticulum, if it is palpable, and other reasons for the patient's complaints.
St-Vil et al. [32]	1991	Establish risk factors affecting complication rate by assessing the patients who underwent excision of the MD due to complications.	Observational Study [164 Patients]	MD discovered incidentally should be resected if ectopic mucosa present or if attached to the umbilicus or to the mesentery by fibrous bands.
Vane et al. [36]	1987	Review of 217 Children with Vitelline Duct Abnormalities	Observational Study [217 Patients]	Elective resection of asymptomatic vitelline remnants in early childhood is sensible at the time of laparotomy for other conditions.

Limitations

The limitations of this study are that only articles in English and German were evaluated, alongside only articles post-1980. Much of the published literature evaluated is from smaller observational studies. Data was lacking about laparoscopy as many studies focused on laparotomies. 

## Conclusions

The management of symptomatic MD has not been nearly as controversial as the management of asymptomatic MD. This literature review worked to illuminate the management of incidental MD. In the literature, it is clear, especially in recent literature, there is a move towards resection for all or in those with high-risk factors. This study was significant in categorizing the literature and evaluating the literature: in terms of patient number, year of publication, and study design. This, in turn, helped to recognize the more recent shift seen in the literature. Research must build upon if resection is recommended for all patients or in those with risk factors. If only in those with risk factors, it is essential to define these risk factors.
